# Analytical validation of a novel multi-analyte plasma test for lung nodule characterization

**DOI:** 10.15761/brr.1000123

**Published:** 2018-11-23

**Authors:** Neil N Trivedi, James K Brown, Tess Rubenstein, Abigail D Rostykus, Amanda L Fish, Heng Yu, Luis Carbonell, Alice Juang, Sandy Kamer, Bhavin Patel, Manpreet Sidhu, Doris Vuong, Shan Wang, Mike Beggs, Alan HB Wu, Mehrdad Arjomandi

**Affiliations:** 1 San Francisco Veterans Affairs Medical Center, USA; 2 MagArray Inc, Milpitas, CA, USA; 3 University of California, San Francisco, USA

**Keywords:** biomarkers, diagnosis, lung cancer, pulmonary nodules, risk models

## Abstract

**Background:**

In the National Lung Screening Trial, 96.4% of nodules had benign etiology. To avoid unnecessary actions and exposure to harm, individuals with benign disease must be identified. We describe herein the analytical validation of a multi-analyte immunoassay for characterizing the risk that a lung nodule found on CT is malignant. Those at lower risk may be considered for serial surveillance to avoid unnecessary and potentially harmful procedures. While those nodules characterized at higher risk may be appropriate for more aggressive actions.

**Objective:**

To validate the analytical performance of multiplexed plasma protein assays used in a novel test for lung nodule characterization.

**Methods:**

A multiplexed immunoassay panel for the measurement of plasma proteins in current smokers who present with a lung nodule on CT scan was evaluated in a clinical testing laboratory. Assay analytical sensitivity, reproducibility, precision, and recovery of Epidermal Growth Factor Receptor (EGFR), Prosurfactant protein B (ProSB), and Tissue Inhibitor of Metalloproteinases 1 (TIMP1) from human EDTA plasma samples were evaluated across multiple runs, lots, and technicians. Interfering substances and sample pre-analytical storage conditions were evaluated for their effect on analyte recovery. The lung nodule risk score reproducibility was assessed across multiple lots.

**Results:**

The assay sensitivities were 0.10 ng/mL EGFR, 0.02 ng/mL ProSB, and 0.29 ng/mL TIMP1 with over three orders of magnitude in the assay dynamic ranges. The assays and analytes are robust to pre-analytical sample handling and the plasma can be stored for up to 4 days at 4°C either when freshy collected or thawed after long-term storage at −80°C. Total imprecision after 20 days of testing remained under 9% for all three assays. Risk score variability remained within a ± 10% risk score range.

**Conclusions:**

The three protein assays comprising the multi-analyte plasma test for lung nodule characterization performed quite acceptably in a clinical laboratory.

## Introduction

Lung cancer is the leading cause of cancer deaths in the United States and worldwide [1]. The high mortality is mainly attributable to its aggressiveness and because most lung tumors are generally detected at advanced, inoperable stages of disease. Despite optimal surgical management, the overall 5-year survival for Non-Small Cell Lung Cancer (NSCLC) remains at only 16.6% [2]. However, if the cancer is detected at an early stage, the 5-year survival exceeds 50% [3]. For this reason, in the last decade, the quest for an effective means of early diagnosis has intensified.

The National Lung Screening Trial (NLST) confirmed in 2011 that early diagnosis of lung cancer can improve survival [4]. Screening for lung cancer in the high-risk group studied in the NLST now has the support of the US Preventive Services Task Force and is recommended by the National Comprehensive Cancer Network Guidelines. However, low-dose Computed Tomography (CT) of the chest for lung cancer screening has significant drawbacks, including cost, radiation exposure, high false-positive rates, and a risk of overdiagnosis of indolent cancers. The results of the NLST have sparked even greater interest in developing more practical and more specific means of early detection of lung cancer, using noninvasive biomarkers of early disease.

A pulmonary nodule on imaging is a common radiographic finding [5]. With improvements in special resolution on CT, the number of patients with pulmonary nodules continues to rise. In the NLST, more than 24% of CT-screened participants had a pulmonary abnormality necessitating further evaluation because of concern for lung cancer [4]. All of these indeterminate abnormalities create an undesirable burden on the healthcare system because each lesion must be evaluated, and most are found to be benign where the prevalence of lung cancer is low.

In the past decade, the characterization of NSCLC into subtypes based on genotype and histology has resulted in dramatic improvements in disease outcome in select patient subgroups. Large initiatives have advanced our understanding of the role of biomarker-driven targeted therapies. In addition, efforts are underway to identify rare genomic subsets through genomic screening, functional studies, and molecular characterization of exceptional responders. Whist these key developments highlight advancements in the treatment of NSCLC, far fewer biomarkers have been demonstrated to characterize the many, often indolent, pulmonary nodules increasingly found on LDCT.

There is an urgent need for a noninvasive test to assist in the characterization of lung nodules in a cost-effective manner at an early stage, when curative interventions are still effective. We have developed and clinically validated a multi-analyte plasma protein assay to help distinguish malignant from benign nodules [reference the clinical validity manuscript in-press]. Here we report on the analytical validation of the multiplexed panel in a commercial clinical laboratory.

## Materials and Methods

### Multiplexed plasma protein assay panel

The multiplexed plasma protein assay panel consisted of immunoassay reagents with antibody pairs specific for Epidermal Growth Factor Receptor (EGFR), pro-Surfactant protein B (ProSB), and Tissue Inhibitor of Metalloproteinases 1 (TIMP1). Antibodies were obtained from R&D Systems, Minneapolis, MN, USA (EGFR and TIMP1) and The Canary Foundation, Palo Alto, CA, USA (ProSB) and were selected based on signal to background levels and compatibility with other reagents in the panel.

The assays were configured as typical immunoassay sandwich assays with one antibody in each pair serving as a capture and the other as detection to be measured with the custom magnetic nanotechnology from MagArray, Inc., Milpitas, CA, USA [6]. The MagArray technology immunoassay reagents consisted of printed circuit boards holding eight MagArray GMR sensor chips spotted with the capture antibodies on individual GMR sensors (80 sensors per chip). Typically, 10 sensors per chip were spotted with each assay-specific capture antibody to provide internal replicates, 40 sensors per chip were spotted with a BSA-based reference protein, and the remaining 10 sensors were left empty. The reference protein sensor signals were used to normalize for chip specific variability in sensor rows and columns, while the empty sensors allowed for assessment of non-specific signal in a clinical sample. The second antibody of each assay pair was labeled with biotin using EZ-Link NHS-PEG4-Biotin from ThermoFisher Scientific, Waltham, MA. The detection signal was generated by custom Magnetic Nanoparticles (MNP) obtained from Miltenyi Biotec, Inc., Auburn, CA, USA to bind to the biotin-labeled secondary antibodies.

The assay protocol was run on a MagArray MR-813 instrument system and included a 90-minute incubation of the GMR chips immersed in the wells of a 96-well microplate containing 1:100 diluted samples, followed by a 1-hour detection reagent incubation. The GMR chips were then immersed in the MNP reagent in the presence of a magnetic field for 20 minutes, during which the GMR signals for each sensor were obtained. Samples were run in duplicate. Analyte concentrations were obtained by transforming the GMR signals though analyte-specific 5-parameter logistic curves. The curves were calculated from testing serially diluted multiplexed assay calibrators containing recombinant proteins, purchased from the same sources as the antibodies, and assigned levels based on the manufacturer’s label claims. Assay validity was defined as replicate wells having a Coefficient of Variation (CV) less than 20%, and human plasma run controls, tested with every assay plate, meeting predetermined concentration values.

### Clinical samples

Human plasma samples for assessing biomarker stability throughout the pre-analytical processing steps were obtained from patients who met inclusion criteria for suspicion of lung cancer and provided informed written consent as part of an IRB-approved study protocol at the San Francisco Veterans Affairs Medical Center, San Francisco, CA, USA. All samples were de-identified and assigned a unique sample ID by the principle investigator so that all laboratory personnel and analysts were blinded to the linkage to protected health information. The 11 subjects participating in this study included four with malignant disease and 7 with benign disease.

Assay run controls were prepared from human plasma purchased from Golden West Biologicals, Temecula, CA, USA. Plasma collected from current smokers and never smokers were screened for levels of the 3 assay proteins to select samples that represent different areas of the assay analytical ranges.

### Biomarker model score calculation

The biomarker model is a Support Vector Machine (SVM) learning algorithm that combines concentration values for each of the three protein biomarkers with clinical health information (age, sex, and lung nodule diameter) to provide the risk of malignancy for a subject. The algorithm is a multidimensional classifier obtained with the e1071v1.6-8 R package a using a linear kernel as the starting point with a tuning function that incorporated 10-fold cross validation to optimize the model cost and gamma parameters to 2.1 and 0.5, respectively. The training set consisted of data from 121 samples (2/3 of the total cohort) randomly selected from the subjects with a malignant lung nodule diagnosis and those with benign disease as indicated on the clinical data record. All subjects were current smokers 25-85 years old with lung nodules measuring 4 to 30 mm in diameter. The prevalence of disease in the training set was 64%. The SVM model output is a score from 0 to 100% that indicates the probability of malignancy for the nodule. A cutoff value of 50% was identified from earlier training and validation studies as the optimal separation between nodules at lower risk from those at higher risk of being malignant [15].

### Pre-analytical sample processing and storage and biomarker stability

To assess the protein biomarker stability throughout the preanalytical process, whole blood was collected from 11 volunteers by venipuncture into standard dipotassium EDTA tubes (Becton Dickenson, Franklin Lakes, NJ, USA) that were centrifuged at 1200g for 15 min to separate the plasma component. The EDTA plasma was decanted into a plastic transport tube and maintained at 2 to 8°C during overnight shipment to the testing laboratory where they were received within 24 hours of collection. Upon arrival, the plasma was divided into 100 μL aliquots for storage at 4 C and −80°C. Duplicate 4°C and frozen aliquots were warmed to room temperature for testing on days 1, 2, and 4 so that one from each of the storage temperatures could then be stored at 4°C for the next test day to evaluate combinations of storage scenarios. Sufficient aliquots were also stored at −80°C to permit testing a freshly thawed aliquot each test day as a reference should the 4°C stored aliquots show consistent changes in biomarker concentrations. A significant biomarker concentration change was identified as a *p* value <0.05 with the mean values and pooled assay standard deviations of the two test conditions being compared. Additionally, the assay run controls were tested along with each sample and time point to be used for monitoring and identifying systematic shifts in assay performance that were independent of the biomarker recovery being assessed.

### Analytical sensitivity

The Limit of Blank (LOB) and Limit of Detection (LOD) were used as indicators of assay sensitivity and the lower limit of quantitation (LLOQ), and were determined by following the Clinical Laboratory and Standards Institute EP-17 guidelines [7]. The LOB was defined as the biomarker concentration at the upper 95% confidence interval of the mean of 16 replicates of the calibrator level zero (sample diluent) tested across 4 plates. The LOD/LLOQ was defined as the lowest level of a plasma sample diluted serially in half, five times (32-fold diluted), that was significantly different from the LOB, with significance defined as non-overlapping 95% confidence intervals.

### Analytical imprecision

The imprecision of the biomarker assays was determined by following the Clinical Laboratory and Standards Institute EP-15A2 guidelines [8]. Four human plasma samples purchased from Golden West Biologicals were tested in duplicate 2-times a day for 20 days to provide 40 replicates for determining components of assay imprecision within run and between runs. Three lots of reagents were included to provide an estimate of the lot-to-lot component of assay imprecision. Assay calibration was set on day 1 and repeated on days 8 and 15 to allow for assay recalibration, should it be needed, as monitored by the assay run controls.

### Analytical linearity and recovery

Biomarker assay linearity was assessed by serially diluting seven clinical samples selected from the algorithm training and testing cohort that contained sufficient biomarker levels to be detectable at a 16-fold dilution after a series of four 1:2 dilutions. Acceptable linearity was defined as a mean percent recovery within 90–110% of the expected value of each 1:2 dilution.

Assay recovery was determined as the repeatable measurement of biomarker levels and an algorithm score for 16 clinical samples across two lots of reagents. The samples were selected from the algorithm training and testing cohort to represent a range of biomarker and algorithm risk scores.

### Interfering substances

The susceptibility of the biomarker assays to typical interfering substances encountered with human plasma samples in the clinical reference laboratory was determined by spiking, into two clinical study plasma samples, bilirubin (conjugated and unconjugated), triglycerides, and hemoglobin at levels up to 5-times expected levels. Interfering substances were obtained from Sun Diagnostics, New Gloucester, ME, USA. The assay susceptibility to biotin interference was also evaluated because of the reliance upon biotin in the assay configuration. Likewise, Human-Anti-Mouse Antibodies (HAMA) were also tested to evaluate their level of interference on the immunoassay format that includes mouse monoclonal antibodies. Samples from individuals with anti-mouse antibody titer were obtained from Sun Diagnostics and tested alone and mixed into two clinical study samples. Purified HAMA was obtained from Zeus Scientific (Branchburg, NJ, USA). An acceptable level of interference was defined as recovery within 20% after adjustment for the endogenous levels of biomarkers in the HAMA sample.

### Score reproducibility

The biomarker model score reproducibility was assessed by calculating the score for clinical samples that were run multiple times in the accuracy study, and for the purchased samples and run controls tested in the precision study.

### Statistical analysis

Data statistical analyses were done using Microsoft Excel version 16.16 and R version 3.4.4. Statistical significance was defined as p-value < 0.05.

## Results

### Pre-analytical processing and biomarker stability

Compared to a freshly collected EDTA plasma sample tested within 1 day after collection, plasma samples stored at 4°C for 2 or 4 days, or frozen at −80°C then thawed and stored at 4°C for 2 or 4 days, show mostly non-significant changes in EGFR, ProSB, and TIMP1 protein levels (Table 1). Overall mean EGFR recovery was 100 ± 7.3% after 4 days storage at 4°C, and 101 ± 7.6% after being frozen and thawed then stored for 4 days at 4°C. Overall mean ProSB recovery was 95 ± 7.9% after 4 days storage at 4°C, and 99 ± 5.8% after being frozen and thawed then stored for 4 days at 4°C. While overall TIMP1 recovery was 97 ± 8.0% after 4 days storage at 4°C, and 99 ± 4.2% after being frozen and thawed then stored for 4 days at 4 °C.

Only two samples showed significant changes in ProSB and/ or TIMP1 levels after 4°C storage for 4 days. Four samples showed significant changes after freeze/thaw and 2- or 4-days storage at 4°C.

Sample EDTA S1 ProSB level dropped to 75% of the day 1 level (*p* = 0.02) and sample EDTA S3 ProSB level dropped to 94% of the day 1 level (*p* = 0.04). Sample EDTA S1 also showed a drop in the level of TIMP1 to 78% of the day 1 level (*p* = 0.03) after 4 °C storage for 4 days.

Sample EDTA S3 showed significant increases in ProSB to 109% (*p*= 0.03) and TIMP1 to 111% (*p* = 0.03) of the day 1 fresh draw values when frozen and thawed then stored for 2 days at 4°C before testing. The same sample aliquots showed similar increased levels after 2 more days of storage at 4°C, however the differences were not significant.

Samples EDTA S8 and S9 each showed a significant 6% drop (*p*= 0.03) in the levels of ProSB or TIMP1, following storage for 2 days at 4°C after being frozen and thawed when compared to the fresh 1 d, 4°C stored aliquot.

### Analytical sensitivity (LOB & LOD)

**Figure 1.**
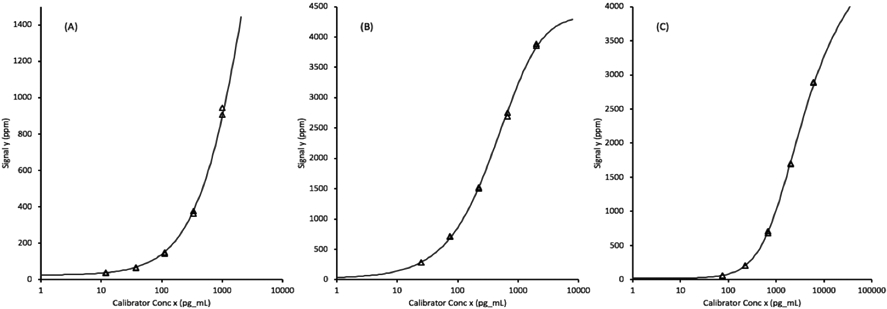
Assay calibration curves fit to 5-parameter logistic functions for (A) EGFR (epidermal growth factor receptor), (B) ProSB (prosurfactant protein B), and (C) TIMP1 (tissue inhibitor of metalloproteinases). The x-axis concentrations units are pg/mL corresponding to a 1:100 diluted plasma sample

Each of the assays were calibrated by testing, in duplicate, assay diluent plus 5 defined levels of recombinant analytes prepared as a multiplexed mixture in the assay diluent. The assay signal levels plotted against the log of the concentrations fit 5-parameter logistic curves that allowed the transformation of unknown sample signals into biomarker concentrations. The quantifiable ranges of the protein biomarkers span approximately 3.5 orders of magnitude (Figure 1).

The Limit of the Blank (LOB) values for each assay, obtained by transforming the upper 95% confidence limits of the signals from 16 replicate of the blank (sample diluent) through the calibration curves, are 0.10 ng/mL EGFR, 0.02 ng/mL ProSB, and 0.29 ng/mL TIMP1, as shown in table 2.

The assay signals obtained from two independent 1:2 serial dilutions of a human plasma sample (assayed in duplicate) are shown in table 3. When diluted 32-fold, the lower 95% confidence limit of the measured concentrations remained above the LOB for all three assays and was selected as the Lower Limit of Quantitation (LLOQ). The assay LLOQ were thus 1.7 ng/mL EGFR, 0.4 ng/mL ProSB, and 2.1 ng/mL TIMP1.

### Analytical imprecision

The imprecision estimates for the 3 biomarker assays from the 40 replicates of 4 human plasma samples tested for 20-days, 2 runs per day by 2 technicians, are shown in table 4.

**Figure 2.**
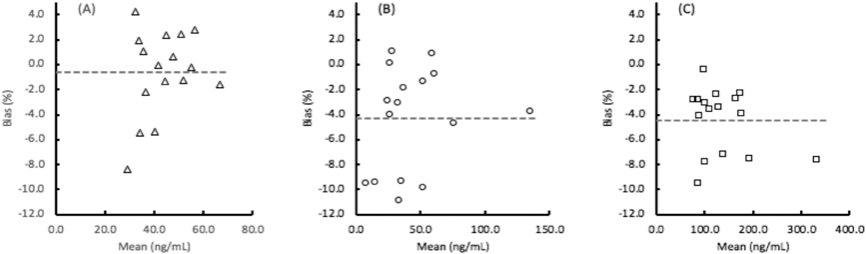
Bias between 2 lots of (A) EGFR (epidermal growth factor receptor), (B) ProSB (prosurfactant protein B), and (C) TIMP1 (tissue inhibitor of metalloproteinases). The mean % bias is the absolute difference of the lot 1 and lot 2 values divided by the mean value. The overall mean values of EGFR: −0.6%, ProSB: −4.3%, and TIMP1: 4.4% are shown as the horizontal dashed lines

The lot-to-lot components of imprecision are also shown in table 4, while the mean lot-to-lot bias for each of the assays were determined to be EGFR: −0.6%, ProSB: −4.3%, and TIMP1: −4.4% as the average percent difference in the concentration values for 16 clinical samples tested with 2 lots of materials (Figure 2).

**Figure 3.**
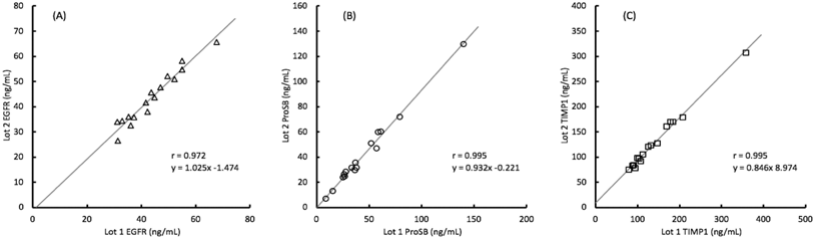
Comparison of analyte concentrations measured by 2 lots of assay materials for 16 clinical samples with the (A) EGFR (epidermal growth factor receptor), (B) ProSB (prosurfactant protein B), and (C) TIMP1 (tissue inhibitor of metalloproteinases) assays

Correlation of the analyte values obtained between each lot were greater than 0.97 as shown in figure 3. The slopes of the correlation line further illustrate the degree of bias between the two lots and show that with EGFR, the lot 2 values are about 2% higher than with lot 1, with ProSB the lot 2 values are about 7% lower than with lot 1, and with TIMP1 the lot 2 values are about 15% lower than with lot 1, although with lower concentration samples, the increased intercept of about 9 ng/mL counteracts some of that shift.

### Analytical linearity and recovery

The mean ratios of the recovered analyte concentrations following serial 1:2 dilutions of seven clinical samples were EGFR: 1.04 ± 0.04, ProSB: 1.09 ± 0.08, and TIMP1: 1.06 ± 0.09 (Table 5). Individual sample recovery ratios ranged from 1.00 to 1.08 for EGFR, 1.03 to 1.14 for ProSB, and 1.02 to 1.14 for TIMP1.

### Interfering substances

Very high levels of the common interfering substances, unconjugated bilirubin, triglycerides, and hemoglobin had less than a 10% effect on the level of the three analytes measured in two human plasma samples compared to the samples without added interfering substances (Table 6). Conjugated bilirubin had a very small effect on the EGFR and TIMP1 recovery, however it decreased the measured level of ProSB by 11%. A high level of triglycerides increased the level of measured ProSB by almost 14%, yet a very high level of triglycerides had a minimal impact. Biotin at very high levels slightly elevated the measured concentrations of the analytes, with ProSB being the most affected with a 9% increase. Likewise, the ProSB levels were the most increased (by 7.8%) with very high hemoglobin, while EGFR and TIMP1 were at most reduced by 4% with very high hemoglobin. HAMA at very high levels of purified antibody caused an increase in all three analytes, with EGFR and TIMP1 being especially affected. Dropping the level of HAMA antibody by just one-half reduced the level of interferences to an acceptable level, as did testing a 1:1 mixture of HAMA serum with the human plasma samples.

### Score reproducibility

**Figure 4.**
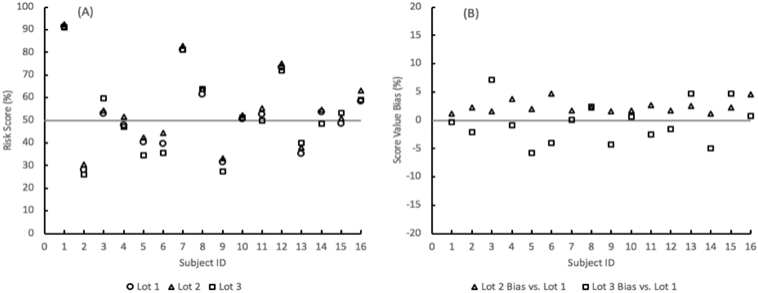
SVM model probability-of-malignancy risk score repeatability for 16 subjects across 3 lots of assay materials. (A) Risk scores. (B) Bias in the score values of material lots 2 and 3 compared to lot 1

The reproducibility of the lung nodule probability of malignancy risk score remained well within a ± 10% bias range between 3 different lots of materials (Figure 4). The risk scores were obtained from the SVM algorithm by inputting the levels of the three analytes and three clinical factors (age, sex, and nodule size) for sixteen subjects from the algorithm training and testing cohort.

In the clinical application of the risk score, the probability of malignancy values is evaluated against a cutoff of 50%, below which the nodule is considered at a lower risk of being malignant. Across the 3 lots of materials, the qualitative risk level provided by the algorithm remained the same for 15 subjects per lot. One subject per lot was within 5% of the cutoff and consequently moved to the other side of the risk cutoff compared to the other 2 lots (Table 7).

**Figure 5.**
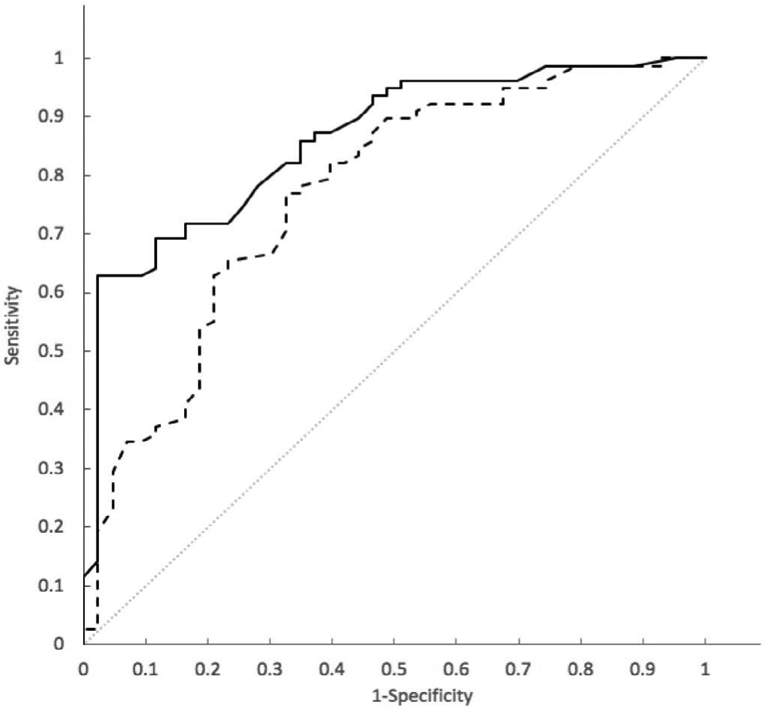
Receiver Operating Characteristic (ROC) analysis of sensitivity vs 1-specifcity for all cut-offs from 0 to 1 for the SVM model (solid line) and the VA model (dashed line) in the model training cohort. The SVM algorithm ROC curve has an area under the curve (AUC) of 0.86. The VA model ROC curve has an AUC of 0.77. The curve of no discrimination for reference is indicated by the gray diagonal line for which the AUC is 0.50

Detailed in a separate publication are the clinical development and validation data of the SVM model as a classifier of indeterminate pulmonary nodules to discriminate between those with a lung cancer diagnosis established pathologically and those found to be clinically and radiographically stable for at least one year. The SVM model for risk classification shows a significant discrimination (*p* = 0.006) of malignant nodules evaluated by Area under the curve (AUC) of a receiver operating characteristic (ROC) curve of 0.86 (95% CI: 0.79-0.93) when compared to the VA model AUC = 0.77 (95% CI: 0.68-0.86) (Figure 5).

## Discussion

The assays in the multi-analyte test for lung nodule characterization were selected from a panel of biomarkers associated with lung cancer tumor progression and thought to have diagnostic and prognostic value [9,10]. Biomarker candidates for which sensitive and specific assays could be developed to measure subtle changes in circulating levels associated with the presence of lung cancer where advanced through the assay development and characterization process. We evaluated the customized assays in early discovery work to measure the biomarker levels in a cohort of subjects from an observational study of PET-CT imaging for lung cancer [15]. Those studies identified that the plasma levels of EGFR, ProSB, and TIMP1 in current smokers were the most informative in assessing the likelihood of malignancy in subjects with an indeterminate lung nodule. Many other biomarkers have been described in the literature as being associated with lung cancer prognosis, although often through tumor tissue gene expression and histological analysis rather than through immunoassays of circulating levels [11-13]. Such proteins may play a pivotal role in lung cancer biology and as such would be biomarkers for the disease, yet they often cannot be reproducibly measured in blood samples due to low levels, poor stability, and/or the lack of specific and reproducible antibodies with which immunoassays can be configured. For those reasons, we selected proteins associated with lung cancer that could be measured reproducibly in blood samples by immunoassays that exhibit requisite sensitivity, dynamic range, and precision. The EGFR, ProSB, and TIMP1 protein assays exhibit sufficient dynamic range to precisely measure the biomarkers from less than 1 pg/mL to at least 6 ng/mL in the 1:100 diluted clinical specimens. The lower limits of the assay measurable ranges are at least 32 times lower than the typical levels found in the clinical samples selected for the assessment of LLOQ. And the imprecision of the assays with the 32-fold diluted plasma sample were at most 6.2% in the case with TIMP1, suggesting that the assay functional sensitivities are even lower. The assay imprecision across 20 days of testing remained well below 10% for the four human plasma samples tested twice each day, further supporting very low functional sensitivities.

Another critical criterion for a successful biomarker test is that an assay be insensitive to preanalytical sample storage variability. Mostly non-significant changes in the measured values were observed with the EGFR, ProSB, and TIMP1 assays with freshly processed samples stored for up to 4 days at 4°C, or frozen within one day of collection and then stored for up to 4 days at 4°C post thaw. Those very few samples which showed a significant difference in the measured protein levels after storage were an exception. Moreover, there were no consistent trends for the changes either with longer storage or across multiple samples, suggesting the apparent changes are more the result of individual testing variability than a systematic or consistent change in the measured analyte levels.

Lot-to-lot assay reproducibility is also critical. It allows the clinical laboratory to have confidence in reporting a risk score based on the protein concentrations, especially when those concentrations are processed through an algorithm with locked coefficients. An analytical bias in the input concentrations can directly influence the reported score. The TIMP1 and ProSB assays exhibited the largest bias between lots of just over 4%, while the EGFR assay demonstrated less than 1% lot-to-lot average bias. The lot-to-lot agreement showed very high correlation when the test samples, as a whole, were compared between the lots. This indicates the lot biases are constant across the range of biomarker values. That consistency was seen when the sample risk scores were obtained for each lot and compared for both bias and risk level reported. With risk scores that ranged from near 20–90%, the maximum difference in risk scores provided by 3 different lots was under 10%. Only 3 samples showed a different risk level due to lot bias and that difference occurred when the small percent difference between the lots fell at the 50% binary cutoff between lower and higher risks.

Assay linearity is an indication of the specificity of the reagents and the degree to which they are free of non-specific interference or binding by endogenous materials found in clinical specimens. The average recovery of concentrations from seven samples diluted through 5 serial 1:2 dilutions was 104 to 109% indicating relatively low non-specific signal issues with the three assays.

Additional specificity and freedom from interference was shown for the typical clinical sample interfering substances of bilirubin, lipids, and hemoglobin at very high levels. At 800 mg/dL hemoglobin, the sample is an unmistakably red solution indicating hemolysis has occurred. Such samples should not be tested, even though the hemoglobin itself won’t interfere, because that level of hemolysis may have changed the biomarker levels from those in non-hemolyzed plasma. Similarly, for samples with high lipid levels, where very little direct assay interference was observed, the elevated fats may reduce the accuracy of pipetting the plasma for the 1:100 dilution.

Biotin at 1200 ng/mL showed no interference despite biotinylated antibody detection techniques being part of the immunoassay format used for these assays. Biotin at such high levels can be observed in clinical specimens from individuals taking very high doses of the B vitamin, and immunoassays need to be free of such interference [14].

## Conclusion

The three protein assays that comprise the lung nodule characterization test show acceptable analytical performance demonstrating the necessary sensitivity, precision, and reproducibility for use in a commercial clinical laboratory. Such performance validates the suitability of these assays to be used to calculate the probability that an indeterminate lung nodule found on CT scan is malignant. When combined with the clinical information of patient age, sex, and lung nodule diameter, the biomarker protein concentrations provide information on the risk of malignancy and help a clinician make a more informed decision about the most appropriate next steps

## Figures and Tables

**Table 1.** Recovery of EGFR, ProSB, and TIMP1 in EDTA plasma samples processed on Day 1 and then stored at 4°C for 2 and 4 days, as well as stored at −80°C and tested after thawing and further storage at 4°C for 2 and 4 days. Significant changes from the Fresh 1d 4°C condition are indicated with a superscripted p value

	Recovery vs. Fresh 1d 4C (%)				
	2d 4°C	4d 4°C	1 F/T	1 F/T, 2d 4°C	1 F/T, 4d°C
**EGFR**					
Sample					
EDTA S1	99	95	101	104	102
EDTA S2	96	96	107	92	93
EDTA S3	99	100	101	104	105
EDTA S4	102	107	102	104	105
EDTA S5	93	88	95	109	113
EDTA S6	101	109	105	109	108
EDTA S7	99	97	97	95	94
EDTA S8	101	108	100	102	101
EDTA S9	101	108	99	104	105
EDTA S10	92	92	97	90	91
EDTA S11	95	96	94	86	90
Mean ± SD	98 ± 3.5	100 ± 7.3	100 ± 3.8	100 ± 7.8	101 ±7.6
**ProSB**					
Sample					
EDTA S1	108	**75 ^p=0.02^**	96	106	105
EDTA S2	95	96	100	95	99
EDTA S3	98	**94 ^p=0.04^**	99	**109 ^p=0.03^**	113
EDTA S4	98	99	100	93	96
EDTA S5	93	89	100	99	103
EDTA S6	97	98	100	92	92
EDTA S7	100	103	96	102	98
EDTA S8	101	101	101	**94 ^p=0.03^**	97
EDTA S9	105	103	100	97	100
EDTA S10	99	91	101	96	97
EDTA S11	99	96	98	96	94
Mean ± SD	100 ± 4.1	95 ± 7.9	99 ± 1.8	98 ± 5.5	99 ± 5.8
**TIMP1**					
Sample					
EDTA S1	98	**78 ^p=0.03^**	92	98	95
EDTA S2	94	99	102	101	102
EDTA S3	97	86	96	**111 ^p=0.03^**	109
EDTA S4	104	101	103	99	99
EDTA S5	95	95	104	99	100
EDTA S6	100	100	102	100	97
EDTA S7	103	103	103	100	100
EDTA S8	100	102	103	99	99
EDTA S9	102	104	100	**94 ^p=0.03^**	96
EDTA S10	96	93	100	95	97
EDTA S11	99	101	97	92	93
Mean ± SD	99 ± 3.1	97 ± 8.0	100 ± 3.8	99 ± 5.0	99 ± 4.2

**Table 2.** Blank sample replicate assay signals and LOB as the upper 95% confidence limit

	EGFR	PROSB	TIMP1
Signal Mean ± SD, PPM (%CV)	16 ± 2.6 (16.3%)	17 ± 2.3 (13.5%)	15 ± 1.4 (9.3%)
Upper 95% CI, PPM	20	21	17
LOB, ng/mL	0.10	0.02	0.29

**Table 3.** LLOQ as the lower 95% confidence limit of a diluted sample above the upper 95% confidence Limit Of the Blank (LOB)

			Conc, ng/mL		
Assay	Dilution	Fold Diluted	Mean ± SD (%CV)	Upper 95% CI	Lower 95% CI
**EGFR**					
	1:100	1	43.7 ± 1.37(3.1%)	46.4	41.1
	1:200	2	22.7 ± 0.50 (2.2%)	23.6	21.7
	1:400	4	12.2 ± 0.26 (2.1%)	12.7	11.7
	1:800	8	6.0 ± 0.07 (1.2%)	6.2	5.9
	1:1600	16	3.5 ± 0.14(4.0%)	3.8	3.1
	1:3200	32	1.8 ± 0.04(2.2%)	2.0	1.7
	Blank	∞	0.0	0.10	--
**ProSB**					
	1:100	1	13.6 ± 0.47 (3.5%)	14.7	12.5
	1:200	2	6.8 ± 0.09 (1.3%)	7.0	6.6
	1:400	4	3.6 ± 0.09 (2.5%)	3.8	3.4
	1:800	8	1.8 ± 0.05 (2.8%)	1.9	1.7
	1:1600	16	1.0 ± 0.04 (4.0%)	1.0	0.9
	1:3200	32	0.5 ± 0.02 (4.0%)	0.5	0.4
	Blank	∞	0.0	0.02	--
**TIMP1**					
	1:100	1	76.6 ± 2.26 (3.0%)	80.5	72.7
	1:200	2	38.7 ± 0.86 (2.2%)	40.0	37.5
	1:400	4	19.6 ± 0.90 (4.6%)	20.9	18.3
	1:800	8	9.5 ± 0.10 (1.1%)	9.6	9.3
	1:1600	16	5.0 ± 0.17(3.4%)	5.4	4.7
	1:3200	32	2.6 ± 0.16 (6.2%)	3.1	2.1
	Blank	∞	0.0	0.29	--

**Table 4.** Assay Analytical Imprecision Summary

	EGFR	ProSB	TIMP1
**Imprecision, %**			
**Intra-assay (within run)**	3.3%	4.1%	2.5%
**Inter-assay (between run)**	7.5%	6.6%	5.2%
**Lot-to-lot**	2.0%	3.0%	3.0%
**Total assay (20 days)**	8.4%	8.3%	6.6%

**Table 5.** Recovery of analyte concentrations following serial 1:2 dilutions

	Sample							
	1	2	3	4	5	6	7	Mean
**Analyte**								
**EGFR**, pg/ml								
Starting	360	434	362	382	456	500	310	
Expected at 1:2	180	217	181	191	228	250	155	
Measured	193	240	188	196	237	252	165	
Ratio	1.07	1.11	1.04	1.03	1.04	1.01	1.06	1.05 ± 0.03
Starting	193	240	188	196	237	252	165	
Expected at 1:2	97	120	94	98	119	126	83	
Measured	101	130	98	105	120	128	87	
Ratio	1.05	1.08	1.04	1.07	1.01	1.02	1.05	1.05 ± 0.03
Starting	101	130	98	105	120	128	87	
Expected at 1:2	51	65	49	53	60	64	44	
Measured	58	70	47	55	64	67	44	
Ratio	1.15	1.08	0.96	1.05	1.07	1.05	1.01	1.05 ± 0.06
Starting	58	70	47	55	64	67	44	
Expected at 1:2	29	35	24	28	32	34	22	
Measured	29	37	23	30	32	34	23	
Ratio	1.00	1.06	0.98	1.09	1.00	1.01	1.05	1.03 ± 0.04
Ratio Mean ± SD	1.07 ± 0.06	1.08 ± 0.02	1.00 ± 0.04	1.06 ± 0.03	1.03 ± 0.03	1.02 ± 0.02	1.04 ± 0.02	1.04 ± 0.04
**Pro SB**, pg/ml								
Starting	1297	649	804	753	184	634	473	
Expected at 1:2	649	325	402	377	92	317	237	
Measured	779	397	454	431	104	357	269	
Ratio	1.20	1.22	1.13	1.14	1.13	1.13	1.14	1.16 ± 0.04
Starting	779	397	454	431	104	357	269	
Expected at 1:2	390	199	227	216	52	179	135	
Measured	494	221	248	255	50	202	144	
Ratio	1.27	1.11	1.09	1.18	0.96	1.13	1.07	1.12 ± 0.10
Starting	494	221	248	255	50	202	144	
Expected at 1:2	247	111	124	128	25	101	72	
Measured	281	124	139	141	26	106	74	
Ratio	1.14	1.12	1.12	1.11	1.04	1.05	1.03	1.09 ± 0.05
Starting	281	124	139	141	26	106	74	
Expected at 1:2	141	62	70	71	13	53	37	
Measured	136	61	69	75	13	51	38	
Ratio	0.97	0.98	0.99	1.06	1.00	0.96	1.03	1.00 ± 0.04
Ratio Mean ± SD	1.14 ± 0.13	1.11 ± 0.10	1.08 ± 0.06	1.12 ± 0.05	1.03 ± 0.07	1.07 ± 0.08	1.07 ± 0.05	1.09 ± 0.08
**TIMP1**, pg/ml								
Starting	1403	3117	1072	1532	1022	1293	2538	
Expected at 1:2	702	1559	536	766	511	647	1269	
Measured	762	1976	601	854	583	722	1551	
Ratio	1.09	1.27	1.12	1.11	1.14	1.12	1.22	1.15 ± 0.07
Starting	762	1976	601	854	583	722	1551	
Expected at 1:2	381	988	301	427	292	361	776	
Measured	443	1135	318	471	290	387	837	
Ratio	1.16	1.15	1.06	1.10	0.99	1.07	1.08	1.09 ± 0.06
Starting	443	1135	318	471	290	387	837	
Expected at 1:2	222	568	159	236	145	194	419	
Measured	235	609	155	230	145	186	418	
Ratio	1.06	1.07	0.97	0.98	1.00	0.96	1.00	1.01 ± 0.04
Starting	235	609	155	230	145	186	418	
Expected at 1:2	118	305	78	115	73	93	209	
Measured	117	321	73	116	69	88	204	
Ratio	1.00	1.05	0.94	1.01	0.95	0.95	0.98	0.98 ± 0.04
Ratio Mean ± SD	1.08 ± 0.07	1.14 ± 0.10	1.02 ± 0.08	1.05 ± 0.07	1.02 ± 0.08	1.02 ± 0.08	1.07 ± 0.11	1.06 ± 0.09

**Table 6.** Mean change in measured analyte level by the addition of potentially interfering substances

		Mean Analyte Concentration Change, %		
Interfering Substance	Level	EGFR	Pro SB	TIMP1
Bilirubin, conjugated	High, 0.2 mg/dL	−2.0	−6.4	−3.2
	Very High, 5 mg/dL	−3.7	−11.1	−4.5
Bilirubin, unconjugated	High, 1 mg/dL	−2.6	−2.3	0.7
	Very High, 15 mg/dL	0.4	0.4	−1.2
Biotin	High, 0.01 mg/dL	2.2	3.3	−0.7
	Very High, 0.12 mg/dL	4.2	8.9	4.1
Triglycerides	High, 150 mg/dL	4.7	13.8	3.7
	Very High, 500 mg/dL	0.7	1.6	−1.4
Hemoglobin	High, 100 mg/dL	−4.0	−5.3	−3.4
	Very High,800 mg/dL	2.9	7.8	1.2
HAMA, serum	50% HAMA serum	2.9	7.8	1.2
HAMA, pure	High, 0.25 mg/dL	7.0	−1.0	4.0
	Very High, 0.5 mg/dL	22.0	9.0	17.0

**Table 7.** Lung nodule risk or malignancy scores and risk levels across three lots of materials. Risk levels in bold italics indicate where the level differs from the other 2 lots

Subject ID	Risk Score, %			Risk Level vs. 50% Cutoff		
	Lot 1	Lot 2	Lot 3	Lot 1	Lot 2	Lot 3
1	91	92	91	Higher	Higher	Higher
2	28	30	26	Lower	Lower	Lower
3	53	54	60	Higher	Higher	Higher
4	48	52	47	Lower	Higher	**Lower**
5	40	42	34	Lower	Lower	Lower
6	40	44	36	Lower	Lower	Lower
7	81	83	81	Higher	Higher	Higher
8	61	64	64	Higher	Higher	Higher
9	32	33	27	Lower	Lower	Lower
10	51	52	51	Higher	Higher	Higher
11	53	55	50	Higher	Higher	Higher
12	73	75	72	Higher	Higher	Higher
13	35	38	40	Lower	Lower	Lower
14	54	55	49	Higher	Higher	**Lower**
15	49	51	53	**Lower**	Higher	Higher
16	58	63	59	Higher	Higher	Higher

## References

[1] Jemal A, Bray F, Center MM, Ferlay J, Ward E, (2011) Global cancer statistics. CA Cancer J Clin 61: 69–90. 21296855 10.3322/caac.20107

[2] Howlander N, Noone A, Krapcho M (2018) Lung and Bronchus Cancer - Cancer Stat Facts

[3] Goldstraw P, Chansky K, Crowley J, Rami-Porta R, Asamura H, (2016) The IASLC Lung Cancer Staging Project: Proposals for Revision of the TNM Stage Groupings in the Forthcoming (Eighth) Edition of the TNM Classification for Lung Cancer. J Thorac Oncol 11: 39–51. 26762738 10.1016/j.jtho.2015.09.009

[4] Aberle DR, Adams AM, Berg CD, Black WC, Clapp JD, (2011) Reduced lung-cancer mortality with low-dose computed tomographic screening. N Engl J Med 365: 395–409. 21714641 10.1056/NEJMoa1102873 PMC4356534

[5] Brandman S, Ko JP (2011) Pulmonary Nodule Detection, Characterization, and Management with Multidetector Computed Tomography. J Thorac Imaging 26: 90. 21508732 10.1097/RTI.0b013e31821639a9

[6] Gaster RS, Xu L, Han SJ, Wilson RJ, Hall DA, (2011) Quantification of protein interactions and solution transport using high-density GMR sensor arrays. Nat Nanotechnol 6: 314–320. 21478869 10.1038/nnano.2011.45 PMC3089684

[7] EP17-A2 (2012) Evaluation of Detection Capability for Clinical Laboratory Measurement Procedures. (2nd Edn). p 80.

[8] EP15-A2 (2014) User Verification of Performance for Precision and Trueness. (2nd Edn). p64.

[9] Hanash S, Taguchi A (2011) Application of Proteomics to Cancer Early Detection. Cancer J 17: 423–428. 22157286 10.1097/PPO.0b013e3182383cab PMC4318261

[10] Taguchi A, Politi K, Pitteri SJ, Lockwood WW, Faca VM, (2011) Lung cancer signatures in plasma based on proteome profiling of mouse tumor models. Cancer cell 20: 289–299. 21907921 10.1016/j.ccr.2011.08.007 PMC3406925

[11] Tang H, Zhao L, Li M, Li T, Hao Y (2018) Investigation of LINC00342 as a poor prognostic biomarker for human patients with non-small cell lung cancer. J Cell Biochem. 10.1002/jcb.27782 30320899

[12] Taube JM, Klein A, Brahmer JR, Xu H, Pan X, (2014) Association of PD-1, PD-1 ligands, and other features of the tumor immune microenvironment with response to anti-PD-1 therapy. Clin Cancer Res 20: 5064–5074. 24714771 10.1158/1078-0432.CCR-13-3271 PMC4185001

[13] Rivas-Fuentes S, Salgado-Aguayo A, Pertuz Belloso S, Gorocica Rosete P, Alvarado-Vasquez N, (2015) Role of Chemokines in Non-Small Cell Lung Cancer: Angiogenesis and Inflammation. J Cancer 6: 938–952. 26316890 10.7150/jca.12286 PMC4543754

[14] CDRH FDA. Safety Communications - The FDA Warns that Biotin May Interfere with Lab Tests: FDA Safety Communication.

[15] Trivedi NN, , (2018) Risk assessment for indeterminate pulmonary nodules using a novel, plasma-protein based biomarker assay. Biomedical Research and Clinical Practice 3: 1–8. 10.15761/brcp.1000173 PMC7480946 32913898

